# The Huntington’s Disease Integrated Staging System

**DOI:** 10.1002/alz.095052

**Published:** 2025-01-09

**Authors:** Jeffrey M Long, Huntington’s Disease Regulatory Science Consortium (HD‐RSC) Work Group

**Affiliations:** ^1^ Departments of Psychiatry and Biostatistics, University of Iowa, IA USA

## Abstract

The Huntington’s Disease Integrated Staging System (HD‐ISS) comprises a biological research definition of HD and evidence‐based staging centered on prognostic biological, clinical, and functional landmarks. It is the result of a formal consensus process within the Huntington’s Disease Regulatory Science Consortium’s Regulatory Science Forum (RSF), a working group made up of expert representatives from industry and academia. The RSF considered prognostic biomarkers, signs, and symptoms of HD, and performed empirical data analysis. It used observational data to calculate healthy‐control‐based landmark variable cut‐offs for stage classification and to internally validate the framework.

The HD‐ISS starts with Stage 0, which comprises individuals with ≥ 40 CAG repeats in the huntingtin gene (HTT) before detectable indications of disease pathology. HD progression is verified by assessments of underlying pathophysiology (Stage 1), then proceeds to a detectable clinical phenotype (Stage 2) and continues to a decline in function (Stage 3). Operationally, individuals can be classified into Stages 1‐3 based on CAG‐independent thresholds of landmark assessments. Importantly, the HD‐ISS covers the entire disease spectrum starting from birth, and is unconstrained by concepts such as “manifest,” “premanifest,” or “prodromal.”

The HD‐ISS allows the definition of study populations across the entire duration of the disease trajectory, including before clinical motor diagnosis. Clinical trials using the HD‐ISS can be implemented immediately. In HD‐ISS Stages 2 and 3, we have knowledge of how to enrich and what endpoints to measure. Although clinical trials in Stage 1 may require further research, that work is ongoing. While participant populations may also be defined by additional enrichment criteria as determined by the study objectives, we propose that all new clinical studies use the HD‐ISS.

